# GINDEL: Accurate Genotype Calling of Insertions and Deletions from Low Coverage Population Sequence Reads

**DOI:** 10.1371/journal.pone.0113324

**Published:** 2014-11-25

**Authors:** Chong Chu, Jin Zhang, Yufeng Wu

**Affiliations:** 1 Department of Computer Science and Engineering, University of Connecticut, Storrs, Connecticut, United States of America; 2 The Genome Institute, Washington University School of Medicine, St. Louis, Missouri, United States of America; Plymouth University, United Kingdom

## Abstract

Insertions and deletions (indels) are important types of structural variations. Obtaining accurate genotypes of indels may facilitate further genetic study. There are a few existing methods for calling indel genotypes from sequence reads. However, none of these tools can accurately call indel genotypes for indels of all lengths, especially for low coverage sequence data. In this paper, we present GINDEL, an approach for calling genotypes of both insertions and deletions from sequence reads. GINDEL uses a machine learning approach which combines multiple features extracted from next generation sequencing data. We test our approach on both simulated and real data and compare with existing tools, including Genome STRiP, Pindel and Clever-sv. Results show that GINDEL works well for deletions larger than 50 bp on both high and low coverage data. Also, GINDEL performs well for insertion genotyping on both simulated and real data. For comparison, Genome STRiP performs less well for shorter deletions (50–200 bp) on both simulated and real sequence data from the 1000 Genomes Project. Clever-sv performs well for intermediate deletions (200–1500 bp) but is less accurate when coverage is low. Pindel only works well for high coverage data, but does not perform well at low coverage. To summarize, we show that GINDEL not only can call genotypes of insertions and deletions (both short and long) for high and low coverage population sequence data, but also is more accurate and efficient than other approaches. The program GINDEL can be downloaded at: http://sourceforge.net/p/gindel

## Introduction

Structural variation (SV) is the genetic variation in structure of an organism's genome. Recent advances in high-throughput sequencing technologies enable the finding of large number of structural variations from population-scale sequence data. For example, the ongoing 1000 Genomes Project [1], [2] has released called structural variations for several human populations from hundreds of sequenced individuals [3]. In this paper, we focus on analyzing low coverage population sequence reads, such as those being generated by the 1000 Genomes Project. There are many types of structural variations. Here, we focus on insertions and deletions (indels), which are more commonly found than other types of SVs in the human genome. The length of indels may vary greatly. In this paper, we focus on indels that are longer than 50 bp, which may potentially have large impact on disease susceptibility.

Over the past several years, a number of increasingly sophisticated computational approaches for discovering indels from sequence reads have been developed. These include e.g. [4]–[8]. Now suppose we have detected indels in the genome. The next natural research question is calling the genotypes of these indels. Genotype plays an important role in genetics. For example, genotypes at some genetic variation sites may affect disease susceptibility. Accurate calling of indel genotypes is not an easy computational problem especially for low-coverage noisy population sequence data. There are several existing approaches for calling deletion genotypes from sequence reads [9]–[11]. One tool is Genome STRiP [9], which has been used in the 1000 Genomes Project. Genome STRiP combines several sources of information contained in the sequence reads. And it is mainly designed for calling genotypes of long deletions. Simulation results show that Genome STRiP can accurately call genotypes of relatively long deletions (200 bp or longer). However, for shorter deletions (i.e. from 50 bp to 200 bp), Genome STRiP performs poorly. Another tool is Clever-sv [11] that is primarily designed for calling genotypes of midsize deletions at medium coverage. Clever-sv combines split-mapped reads and discordant paired-end reads to call genotypes of deletions. Although Clever-sv works well for intermediate deletions, it performs less well for low coverage data. Dindel [12] can also call genotypes of deletions, but it mainly focuses on small deletions (30 bp or smaller). Pindel [5] is primarily designed for calling insertions and deletions. Recently, a new option has been added to Pindel for calling genotypes for both short and long deletions. However Pindel is quite sensitive to reads coverage, and performs poorly when the coverage is low.

In this paper, we develop a new indel genotype calling approach (called GINDEL) from low coverage population sequence reads that not only works for insertions and both short and long deletions, but also is more accurate and efficient than Genome STRiP, Pindel, and Clever-sv. In particular,

1. Similar to Genome STRiP and Clever-sv, GINDEL also extracts various information (called features) from sequence reads and calls genotypes by combining these features. A main difference is that GINDEL uses more features than Genome STRiP and Clever-sv. Another difference is that GINDEL uses a machine learning approach for combining features. As a result, GINDEL uses the data in a more effective way. Also, GINDEL can call insertion genotypes, while the current approaches only call deletion genotypes.
2. We show that GINDEL outperforms Genome STRiP, Clever-sv, and Pindel in both the genotype accuracy and also efficiency on both simulated data and real population sequence data.

## Background

### Signatures of indels on reads

Each indel can be specified by a pair of breakpoints. A breakpoint is a genomic coordinate in the reference genome, which indicates where the indel starts or ends. When sequence reads are taken from an indel site, various signatures left by the indel may be found from the reads.

In this paper, we are mainly concerned with paired-end reads. Suppose a paired-end read (also called read pair) is mapped to the reference genome using a tool such as BWA [13]. Sometimes both reads of a read pair are mapped with the two reads agreeing on the orientation and order, but the insert size is discordant with the library insert size. This can be an indication that there is a SV (e.g. deletion) between the two reads since the chance of having unusually large or small insert size is small. So we have:

**(i) Discordant encompassing pair signature**: the number of discordant pairs. A discordant pair has insert size from the two mapped reads is outside the range 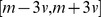, where 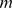 is the mean insert size and 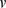 is the standard variation of the insert size.

**Figure 1:**
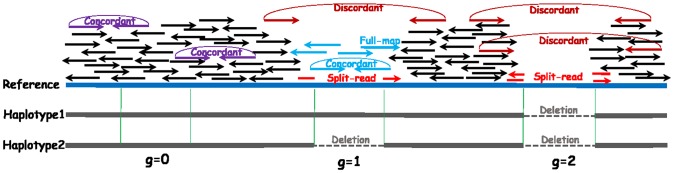
Features for the three deletion genotypes: discordant and concordant pairs, split-read, fully-mapped reads, and read depth.

See Figure 1 for an illustration of discordant encompassing pairs for deletions. There are many methods that detect SVs by analyzing the insert size of discordant encompassing pairs, such as PEMer [14], BreakDancer [4] and VariationHunter [15].

Now we consider unmapped reads. Often unmapped reads are caused by sequencing error. Another cause of unmapped sequence reads is the presence of structural variations such as indels. To fix ideas, let us consider the case of deletions. When a read contains the breakpoints of a deletion, the read will contain two parts: one from the region prior to the deletion and one from the region following the deletion. We call such a read spanning read for the deletion. The spanning read may be unmappable as one piece because the read is a concatenation of the two parts and is not contained in the reference genome as a whole. Sometimes one or both parts of a spanning read can be mapped to the reference genome. If both parts are mapped correctly, the mapped spanning reads may reveal the position of the deletion. So we have:

**(ii) Split-read signature**: the number of split-reads. A split-read is a read that cannot be mapped as a whole but can be mapped as two parts.

Figure 1 gives examples of mapped split-reads. Split-reads mapping has been used in the programs Pindel [5], SVseq [6], [7] and PRISM [8] for finding deletions.

Read depth is another commonly used signature for finding deletions. For a position in the reference genome, read depth is equal to the number of mapped reads that cover that position. Intuitively, when a deletion is present, the average read depth of the deletion region is likely to be smaller than expected. So we have:

**(iii) Read depth signature**: the average read depth of the deletion region. A region on the reference with smaller read depth than expected may suggest the presence of the deletion. Average read depth of a region is calculated by: 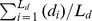, where 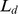 is the length of the region and 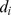 is the read depth at position 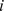 of the region.

Figure 1 illustrates the difference in read depth for different deletion genotypes. In particular, when deletions are present, read depth tends to be smaller. Read depth has been used in programs such as CNVnator [16].

### Genotypes and reads

A diploid organism (such as human) has two copies for each chromosome. Although these are often called ‘copies’, they are not identical. We consider a genetic variation site (such as a deletion). For one copy of chromosome, we use state (called allele) 1 to indicate that the variation is indeed present at this copy, and use state 0 otherwise. For convenience, we often refer to allele 0 as the wild-type and allele 1 as indel allele. A description of the conflated (mixed) data from the two copies is called a *genotype*. When both copies have allele 0 (respectively 1) at a site, the genotype has state 0 (respectively 2), and is called a homozygote. Otherwise, the genotype has state 1 at that site and is called a heterozygote. Note that a genotype is for a particular diploid individual at a specific variation site.

In this paper, we want to call the genotypes of indels from low coverage population sequence reads. Accurate calling of indel genotypes from low-coverage whole-genome sequencing data poses a significant challenge. First, current population sequencing usually adopts the low coverage scheme [1], [2]. So the average coverage (i.e. the number of reads overlapping the sites of interests) is usually low. Moreover, sequence data is obtained using the so called “shotgun” approach. That is, short DNA fragments called reads are generated from randomly selected locations on the two chromosomes. There is usually large variations in coverage. Some genetic variation sites may have very low coverage or even no reads at all. Even if there are some reads at a site, calling genotypes may still not be easy. Note that a read may be from either chromosome copy. If, for example, there are only two reads generated from a heterozygous deletion locus, there is a 50% chance that one allele (say allele 1, the one with deletion) would be missed. If this happens, it is difficult to reliably call the heterozygote genotype. Other inherent noises (such as sequencing errors) will only make matters worse.

A simple way of calling genotypes using sequence reads is to first obtain the number of reads showing each type of allele, and then call the genotypes based on these read counts. Such a scheme has been used in calling SNP genotypes from sequence reads (see e.g. [17]). Our experience is that this simple scheme is not very accurate for our problem. A main problem with this scheme is that it does not consider all the information about indels. Recall that there are different signatures (e.g. spanning reads and discordant encompassing pairs) for an indel. It is unclear how to combine these information from different sources when calling genotypes. There are several existing computational approaches for calling indel genotypes, including Genome STRiP [9], Clever-sv [11], Pindel [5] and Dindel [12]. Genome STRiP uses encompassing pairs and read depth (but not split-reads) and calls deletion genotypes based on Bayesian likelihood. Empirical results show that Genome STRiP performs well for longer deletions (1,000 bp or longer). For short deletion (less than 200 bp), Genome STRiP often does not call genotypes and only reports “low quality”. Clever-sv is a tool for calling genotypes of deletions. In addition to using features such as split-reads, Clever-sv also uses Mendelian rules in genotype calling. Originally developed in [5] for the purpose of finding deletion sites, Pindel has been extended to call the genotypes of indels. Simulation results show that both Clever-sv and Pindel do not perform well for low coverage data. Dindel only calls genotypes for indels that are shorter than the ones we are focusing in this work. This indicates that there is still much room for improvement in indel genotype calling.

## Methods

### High-level approach

**Figure 2:**
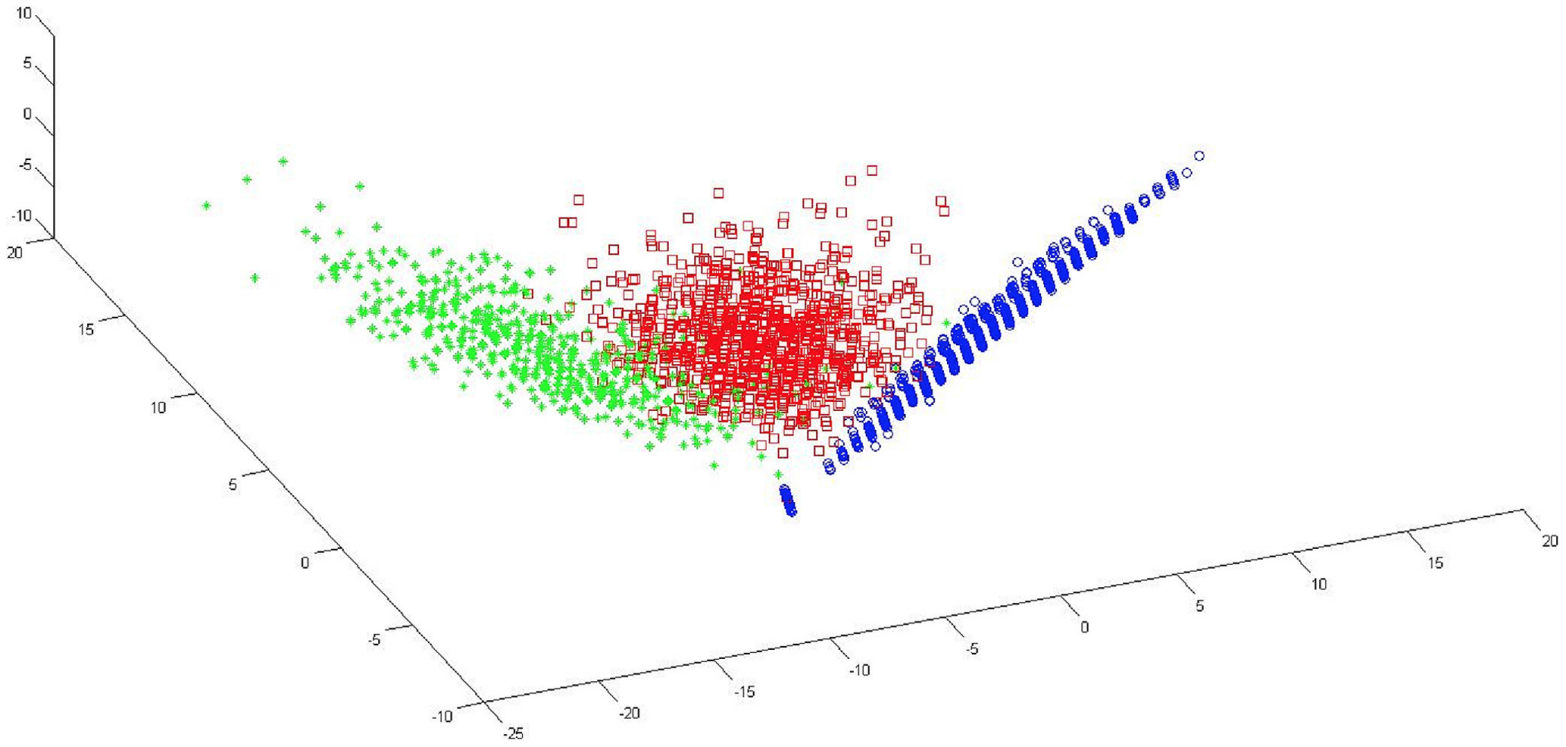
Illustration of classification-based genotype calling with the real 1000 Genomes Project data. Each point refers to one of the 4096 deletion genotypes for 123 deletions on chromosomes 11 and 20. The coordinates of the points are calculated based on extracted features, and each point is labeled by the benchmarked genotype [18]. For better visualization, the number of features used is reduced. There are clearly three categories for the benchmarked genotypes, where each category corresponds to one of three possible genotypes: 0 (circle/blue), 1 (square/red) and 2 (cross/green).

Our method, GINDEL, assumes that a list of indels (including the types and genomic positions) and a set of population sequence reads in the BAM format are given as input. GINDEL also requires training data (that is, known indel genotypes for the indels along with the sequence reads). The key idea of GINDEL is treating each genotype for each given indel and each individual as a data point in a multi-dimensional space, and calling genotypes based on classification of these points. The coordinates of these data points are based on features collected from sequence reads from the specific individual and indel. Choosing the appropriate features is the key for GINDEL. If an extracted feature can represent the property of one aspect of the whole data, then this feature is considered as a good feature. In contrast, if an extracted feature can only represent the property of part of the data, then we see this feature as a bad or less important feature. GINDEL uses the three signatures explained in the Background Section as features and several more features for the presence of indels. It is important to note that genotype calling requires features indicating both the presence *and* the absence of indels. GINDEL thus also uses several features for showing the absence of indels. Moreover, features for insertions and deletions may be different. Finally, GINDEL combines these features and views each genotype of an indel as a point in a multi-dimensional space. The dimension is equal to the number of features we use for the genotype. Then the genotyping problem becomes a classification problem. That is, the genotypes classified into the same category are called the same genotype states. Figure 2 illustrates the classification approach using 4096 deletion genotypes from the real 1000 Genomes Project data [18]. There are clearly three categories for these points, which correspond to the three genotypes: 0, 1 and 2. We use support vector machine (SVM) to classify the genotypes. We first train the SVM with some training data. Then with the trained SVM, GINDEL can call the genotypes of deletions and insertions accurately and efficiently.

### Details of our approach

GINDEL calls genotypes of indels in three steps. In the first step, GINDEL collects all the relevant reads for each given indel from the given reads in the BAM file. Then we collect all the features needed by parsing these relevant reads of each individual for the indel. Finally, we train an SVM model using the training data, and then use the trained SVM model to call the genotypes of testing data.

#### Reads collection

For a given deletion or insertion with known breakpoint 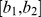, GINDEL collects all the reads whose mapping positions are relevant to this indel. We say a read is relevant to the indel at 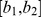 if the read falls within the region 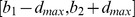, where 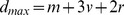. Here, 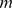 and 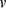 are the mean and standard deviation of the insert size in the given reads, and 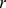 is the read length. GINDEL allows the user to specify the values of 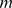 and 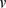. If 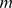 and 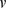 values are not specified, GINDEL calculates 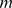 and 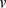 from the reads in the BAM file. Intuitively, 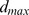 specifies the maximum distance between a read and the boundary of the indel if the read pair containing this read overlaps the indel. We say a read pair overlaps the indel if the interval formed by the leftmost and rightmost positions of the read pair overlaps the interval of the indel. If the insert size of a paired-end read is larger than 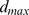, we treat this pair as an invalid pair (possibly wrongly mapped), which is then discarded. We also discard low-quality reads from the set of the relevant reads. A paired-end read is discarded if both reads are of low mapping quality or unmapped. A read may have multiple alignments in the BAM file, and this is allowed in our approach. Thus, all surviving reads are mapped or partially mapped. There are three allowed situations for a surviving paired-end read: (i) both mapped, (ii) one end mapped and the other end partially mapped (see below), and (iii) one end mapped and the other end unmapped.

#### Feature parsing

Once relevant sequence reads are found, GINDEL parses various features at an indel of an individual. This is the key step in our approach. As explained before, we use features for showing both the presence and absence of indels. These features are obtained by analyzing three aspects of reads for an indel: (i) encompassing read pairs (i.e. two reads in the pairs are mapped to different sides of the indel), (ii) spanning reads (i.e. reads mapped to a region that overlaps the indel), and (iii) read depth near the deletion. Table 1 lists all the features used by GINDEL for insertions and deletions.

**Table 1:** List of features used to call genotypes of insertions and deletions.

Features	Deletion	Insertion
Discordant and concordant pair-end reads	Yes	Yes (for short insertion)
Single-end-mapped pair-end reads	No	Yes (for long insertions)
Partially-mapped spanning reads	No	Yes
Fully-mapped reads	Yes	Yes
Split-reads	Yes	No
Read depth	Yes	No

Yes/No: whether a feature is used in calling the genotypes of the indels.

**Encompassing pairs.** We first find all discordant encompassing pairs for the indel. Note that this is made easy by the known breakpoints of the indel: the insert size of an encompassing pair for an indel should be close to the expected library insert size after being adjusted by the indel length. Moreover, concordant encompassing pairs suggest at least one wild-type allele. So we have:

**(iv) Concordant encompassing pair feature**: the number of encompassing pairs that have insert size from the mapped positions that falls within the range 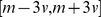. A high value of this feature shows the presence of at least one wild-type allele.

Recall that 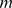 is the mean and 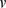 is the standard deviation of the insert size. See Figure 1 for an illustration of concordant pairs. The case of insertion needs more consideration. Note that if the insertion is very long, then it is possible that one end will be from the inserted sequence and will be unmapped. So we may not be able to find any discordant encompassing pairs for an insertion. Here, we can extract another feature from paired-end reads:

**(v) Single-end-mapped pair feature**: the number of read pairs that one read is mapped and the other is unmapped. This type of read pairs may occur when one read originates within the inserted sequence and the other read which is outside the insertion. So a high value suggests the existence of at least one long insertion allele.

**Spanning reads.** GINDEL uses split-read signature. In addition, GINDEL also uses partially-mapped reads:

**(vi) Partially-mapped reads feature**: the number of spanning reads that cannot be mapped as a whole but one segment (prefix or suffix) is mapped (as soft-clip). A high value of this feature suggests the existence of an indel allele.

A partially-mapped read differs from a split-read in that both prefix and suffix segments of a split-read can be mapped. Partially-mapped reads can be useful when split-reads cannot be found. For insertions, partially-mapped reads are more useful because one segment of a spanning read is from the inserted sequence and thus cannot be properly mapped. Besides split-reads and partially-mapped reads (which are unmapped reads as a whole), we also have:

**(vii) Fully-mapped spanning reads feature**: the number of spanning reads that are mapped as a whole. A high value of this feature suggests the existence of a wild-type allele.

For example, in the g = 1 case of Figure 1, the presence of fully-mapped reads supports the existence of a wild-type allele. Note that features obtained from split-reads or partially-mapped reads are more sensitive to the accuracy of breakpoints than other features. In practice, sometimes SV discovery tools may not be able to call the breakpoints with high accuracy. Also, reads may not be perfectly clipped at the breakpoints because of error or high sequence similarity near the breakpoints. So we allow some flexibility in determining whether a split-read or partially mapped read occurs at the specific breakpoint of a given indel. GINDEL provides a “-c” option with the default value being 15, which means reads clipped 15 bp from the breakpoints are considered as clipped at the breakpoints.

**Read depth.** Read depth is informative for deletion genotype calling because the number of reads mapped to the region of the reference genome between the two breakpoints depends on the deletion genotype. For homozygous deletions, no or very few reads are to be mapped to this region and thus the read depth is close to 0. The read depth for heterozygous deletions is likely to be between the two homozygous cases. Another advantage of read depth is that it does not rely on knowing the exact breakpoints. GINDEL uses the read-depth signature for evidence for the presence of both the deletion and wild-type alleles. Refer to Figure 1 for an illustration.

We note that coverage may be quite different in training and testing data. Even when training data and testing data are from the same source, coverage around different deletions or insertions may be different. Suppose the coverage around one deletion is low. Then even if there are only few discordant encompassing pairs, these reads still provide a strong signal that the deletion is not homozygous wild-type. However, for another deletion whose local coverage is high, a small number of discordant encompassing pairs may not be a strong signal. Therefore, we need to normalize the features parsed for each indel. GINDEL uses local read depth to do normalization for each indel. Here, local read coverage is equal to the average read coverage of two regions 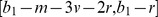 and 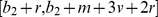, where 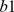 and 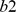 are the breakpoints of the indel (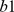 and 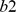 are same for insertions), 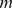 and 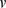 are the mean and standard deviation of insert size, and 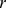 is the read length.

#### Model training and genotype calling

All features are quantified using the sequence reads for the specific individual and the indel for all individuals. Let 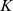 be the number of features for a specific type of indel (say deletion). We obtain a length-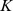 vector. We treat each such vector as a point in a 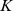 dimensional space. There are total 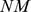 points, where 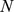 is the number of diploid individuals and 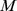 is the number of deletions. Intuitively, points with the same genotype values tend to resemble each other. So, we expect to see three categories of points, which correspond to three possible genotypes: 0, 1 and 2. Refer to Figure 2 for an illustration. We use the support vector machine to perform classification. In particular, we use LibSVM [19] to train models using provided training data, and then use the trained SVM model to call genotypes of test data. The model training usually takes two steps. First, all training data is labeled and then scaled. When there is no validated data or labeled data provided, we can use simulated data to train the model. A simulator is released in our program. See the Material S1 for detail steps of generating simulated data. Second, grid search and 10-cross validation are used to find the optimal parameters, and default kernel function is used in this procedure. Then we use the trained model to call the genotypes of the test data. Also for training and testing, a “-b” option can be used in LibSVM to generate the probability for each genotype.

## Results

GINDEL can call genotypes for both insertions and deletions. We test GINDEL on both simulated and real data for calling insertion and deletion gentoypes. For insertion genotype calling, we cannot find any benchmarked real data, nor existing tools for calling genotypes of insertions (larger than 50 bp). Thus, we use an indirect measure for evaluating the accuracy of GINDEL. To evaluate the performance of GINDEL on deletion genotype calling, we compare GINDEL with the latest version of Genome STRiP (version 1.04.1358), Pindel (version 0.2.5), and Clever-sv (interim version downloaded at 05/01/2014) on both simulated data and real data. Here, we present the key results. More detailed results, commands used and detailed parameters are given in the Material S1.

### Calling deletion genotypes with simulated data

#### Results for different deletion sizes and coverages

45 individuals are simulated from chromosome 15 of NCBI36. We introduce 221 deletions from the CEU population reported by [3] to the simulated datasets. The deletion file is union.2010
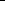
06. deletions.genotypes.vcf.gz in the 1000 Genomes Project data release. For these deletions, the smallest, largest and average deletion sizes are 51 bp, 160,798 bp and 339 bp respectively. We choose these called deletions because these deletions include both long and short ones. Because the haplotypes are unknown, for heterogeneous deletions we arbitrarily put one copy of the deletion on one of the haplotypes of an individual. We simulate paired-end reads with the reads simulator wgsim, with 2% error rate, read length of 100, and insert size of 500. Three datasets with coverage 4.2×, 6.4×, and 10× are simulated. BWA is used with default parameters to map the simulated reads to NCBI36.

We run GINDEL, Genome STRiP, Pindel, and Clever-sv on this data. For GINDEL, to make sure the results are convincing, 10 rounds of experiments are done. For each round, we randomly choose 31 individuals (75% of all the individuals) as training data, and the other 14 individuals (25% of all the individuals) are used as testing data. The average accuracy of the 10 rounds is reported. For Genome STRiP, there are various parameters for users to set. One parameter is called depth.effectiveLengthThreshold, which specifies the minimum effective length of deletions for calling genotypes. The default of this parameter is 200. We use the following two ways to run Genome STRiP. The first is using the default setting (denoted as STRiP v1), which does not call deletions shorter than 200 bp. The second is setting the effective length threshold to be 51 bp (denoted as STRiP v2). This setting allows calling more deletion genotypes longer than 51 bp. We note that Genome STRiP does not call genotypes for all deletions longer than the effective length threshold (some are marked as low quality and not called). For Pindel, we first run with default parameters, and then use pindel2vcf to call out the genotypes. Not all of the benchmark deletions are called out in the first step. So we only use those deletions that overlap with the benchmark in comparison. For Clever-sv, because it does not accept the alignment generated by BWA, we first run laser (a tool used in Clever-sv for reads alignment), then run genotyper (a tool used by Clever-sv to call genotypes). For laser, “-w” option with value “ten percent of the read coverage” is used. And for genotyper, default parameters are used.

Table 2 shows the results of GINDEL, Genome STRiP, Pindel, and Clever-sv for calling deletion genotypes on simulated data with three different coverages. To evaluate the effects of sequence coverage and deletion length, we provide itemized results for various deletion lengths besides the overall accuracy for each coverage. For longer deletions (1.5 kbp or longer), the gap between GINDEL and Genome STRiP is smaller. For shorter deletions, GINDEL tends to significantly outperform Genome STRiP. This is especially true for deletions of length 400 bp or shorter, where there are totally 116 such shorter deletions (about 52.5% of all deletions). For Pindel, no genotypes are called for coverage 6.4× and 4.2×. For the dataset with coverage 10×, 47,457 deletions are called out, where only 104 deletions are concordant with the benchmark. Both GINDEL and Clever-sv perform better for intermediate deletions (length 200–1500 bp) than Genome Strip and Pindel. GINDEL performs better than Clever-sv for larger (1.5 kbp or longer) and smaller (50–200 bp) deletions, especially for datasets of coverage 6.4× and 4.2×. Overall, GINDEL performs well on all three coverages compared with the three existing tools.

**Table 2:** Comparison of GINDEL, two versions of Genome STRiP (with different setting of the effective length threshold), Pindel and Clever-sv on simulated data.

Coverage	Length	Del. Num.	GINDEL	Genome STRiP v1	Genome STRiP v2	Pindel	Clever-sv
10×	50–200	70	0.9869	0.0000	0.6254	0.5476	0.9857
	200–400	46	0.9769	0.4423	0.8349	0.5502	0.9859
	400–500	13	1.0000	0.8516	0.9327	0.3333	1.0000
	500–1500	34	1.0000	0.9942	0.9942	0.5830	0.9948
	>1500	58	0.9979	0.9805	0.9805	0.5950	0.9540
6.4×	50–200	70	0.9791	0.0000	0.5679	-	0.8867
	200–400	46	0.9697	0.3927	0.7869	-	0.9754
	400–500	13	1.0000	0.8496	0.9162	-	0.9915
	500–1500	34	1.0000	0.9542	0.9542	-	0.9699
	>1500	58	0.9967	0.9548	0.9667	-	0.9345
4.2×	50–200	70	0.9645	0.0000	0.4429	-	0.6756
	200–400	46	0.9651	0.3562	0.6984	-	0.9068
	400–500	13	1.0000	0.8308	0.8940	-	0.9538
	500–1500	34	0.9979	0.9144	0.9144	-	0.9458
	>1500	58	0.9963	0.9686	0.9801	-	0.8966

Results on the percentage of correctly called genotypes are given for deletions with different lengths. Del. Num. indicates the number of deletions in specific length (the number of genotypes is Del. Num. multiply the number of individuals), and because Pindel discovers 104 out of the 221 deletions, so the number of deletions in each specific length (from small to large) are: 24, 21, 5, 20, and 34 respectively. Pindel does not call genotype for data with lower coverages. So no results are reported.

#### Results for different features

GINDEL uses a machine learning approach combing five features to call the genotypes of deletions: the number of discordant read-pairs, the number of concordant read-pairs, the number of split-mapped reads, the number of fully-mapped reads, and read depth. In order to see how different features contribute to the final results, we combine different features that are generated by GINDEL. We train a model for each group of features and test the accuracy of each combination. The same 6.4× dataset used in the last section is used in this experiment. And we randomly choose 75% of the data as the training data, and the rest 25% as the testing data. Table 3 shows the eight groups of results of combining different features. The results show that: 1) The number of split-mapped reads and the number of fully-mapped reads contribute more for short deletions (50–200 bp), and they also are good features for larger ones. 2) The number of discordant read-pairs and the number of concordant read-pairs have more effects for large deletions (larger than 200 bp). 3) Read depth performs less well when used independently. But the classifier will performs better if read depth is combined with other features, which indicates read depth is also a good supplementary. 4) All the five features have significant contributions to the final results. The more features are combined, the better the performance of classifiers.

**Table 3:** Accuracy of GINDEL trained by combination of different features.

Length	Gntps	D. C.	S. F.	R.	D. S.	D. C. R.	R. S. F.	D. C. S. F.	All
50–75	294	0.8844	0.9218	0.7721	0.9252	0.8844	0.9082	0.9388	0.9592
75–100	294	0.8878	0.9728	0.8844	0.9490	0.9354	0.9728	0.9762	0.9796
100–150	238	0.8866	0.9958	0.8277	0.9454	0.9664	0.9874	1.0000	1.0000
150–200	154	0.9351	0.9610	0.9351	0.9870	0.9870	0.9545	0.9870	0.9870
200–300	224	0.9420	0.9200	0.8259	0.9464	0.9688	0.9375	0.9643	0.9688
300–400	420	0.9262	0.9333	0.8262	0.9381	0.9619	0.9405	0.9643	0.9667
400–500	182	0.9615	0.9670	0.8736	0.9560	1.0000	0.9780	1.0000	1.0000
500–800	140	0.9929	0.9714	0.9214	0.9929	1.0000	0.9714	1.0000	1.0000
800–1100	168	1.0000	0.9464	0.8929	1.0000	1.0000	0.9583	1.0000	1.0000
1100–1500	168	0.9702	0.9643	0.9226	0.9702	1.0000	0.9702	0.9940	1.0000
1500–2000	210	0.9714	0.9762	0.9476	0.9857	1.0000	0.9762	0.9952	0.9952
2000–4000	252	0.9762	0.9444	0.9246	0.9722	0.9960	0.9841	0.9881	0.9960
4000–9000	238	0.9748	0.9500	0.8277	0.9706	0.9748	0.9538	0.9832	0.9874
>9000	112	0.9821	0.9821	0.8660	0.9821	1.0000	0.9821	1.0000	1.0000

Results on the percentage of correctly called genotypes are given for deletions with different lengths. Gntps represents the total number of genotypes in specific length. D., C., S., F., and R. represents # of discordant read-pairs, # of concordant read-pairs, # of split-mapped reads, # of fully mapped reads, and read depth. All means using all the five features.

#### Results for different deletion frequencies

Because GINDEL treats each deletion independently, GINDEL can call the genotypes of deletions for both single individual and multiple individuals in a population. To see how GINDEL works on population data, we also give the genotype accuracy of deletions with different deletion frequencies. We use the same three simulated datasets of coverage 10.0×, 6.4× and 4.2×. Here we do not compare with Genome STRiP and Clever-sv. This is because Genome STRiP is primarily designed for long deletions, while Clever-sv is mainly designed for midsize deletions. If we calculate statistic according to deletion frequencies, it will be unfair for these tools, because for one specific deletion frequency, there may be short, midsize and long deletions. Indeed our experiment also shows that the accuracy for some specific deletion frequencies is really low for Genome STRiP and Clever-sv. So here we only show the results of GINDEL. Table 4 shows the performance of GINDEL on different deletion frequencies with different coverages. The results show that GINDEL performs well for different deletion frequencies with different coverages. The accuracy drops a little as deletion frequency increases. This indicates that GINDEL performs a little better for homozygous wild-types than the other two kinds of genotypes.

**Table 4:** Performance of GINDEL on different deletion frequencies with different coverage.

Coverage	Del. Freq.	Del. Num.	GINDEL
10.0×	0–5	138	0.9984
	6–15	34	0.9895
	16–30	27	0.9630
	31–45	34	0.9675
6.4×	0–5	138	0.9974
	6–15	34	0.9790
	16–30	27	0.9444
	31–45	34	0.9416
4.2×	0–5	138	0.9964
	6–15	34	0.9748
	16–30	27	0.9418
	31–45	34	0.9351

Del. Freq. is the deletion frequency from 0 to 45 (there are 45 individuals in all). Del. Num. is the number of deletions on specific deletion frequency.

### Calling deletion genotypes with real data

Three datasets, high resolution CNV benchmark data, low coverage sequence data with short deletions, and high coverage trio data, are used as real data. We first compare GINDEL and Genome STRiP using the high resolution CNV benchmark data, low coverage sequence data. Here, we do not compare with Clever-sv because Clever-sv requires raw reads (in the fastq format), but not all reads are released in these two datasets. We also do not compare with Pindel because the output of Pindel changes significantly for different coverages, and this makes the comparison complex and unfair. In order to compare with Clever-sv and Pindel, we perform a separate experiment using the high coverage trio data. In this second experiment, we do not compare with Genome STRiP because Genome STRiP requires at least 20 to 30 individuals to get good results.

#### High resolution CNV benchmark data

We first use the low-coverage population sequence phase two data released by the 1000 Genomes Project. This data consists of alignment files of 66 individuals on chromosomes 11 and 20 (33 individuals for each chromosome). For the 33 individuals of each chromosome, 13 of the individuals are from CEU population and the others are from the YRI population. These alignment datasets are mapped using BWA with soft-clips on NCBI37. There are 122 deletions in these two chromosomes, where genotypes are called in [18]. These CNVs are validated through array-CGH using a set of NimbleGen, and thus we use these genotypes as our benchmark. These deletions are usually long: only 16 are within the range of 450 bp to 960 bp, and the others are longer then 1 k bp. The minimum deletion size is 450 bp. The maximum deletion size is 88,384 bp, and the average size is 2,461 bp.

Recall that GINDEL needs training data. There are various ways of training. One approach is, for each chromosome, splitting the collected feature data into training data and testing data. Here, we perform 10 rounds experiments to get convincing results. For each round, we randomly choose 75% of all the individuals as the training data and the rest 25% as the testing data. Table 5 shows the average accuracy of the 10 rounds on the benchmark data. To evaluate GINDEL and Genome STRiP for data with different coverages, we manipulate the reads by randomly keeping a fraction of the reads in the original data. The fractions are chosen from 100% (i.e. using all the reads), 75%, 50% and 25%. Training strategies may also affect the genotype accuracy. We experiment with two other training strategies: (i) train with given genotypes on one chromosome (say chromosome 11), and use the trained model to call genotypes on the other chromosome (say chromosome 20); (ii) train with simulated data, and test on real sequence reads. It is clear that GINDEL consistently outperforms Genome STRiP on all the tests we performed on these longer deletions. Also note that training does affect the genotype accuracy. Genotypes called with models trained by simulated data tend to be slightly less accurate than trained by real data.

**Table 5:** Comparison of GINDEL and Genome STRiP on high resolution benchmark data with various coverages on chromsomes 11 and 20.

		GINDEL		Genome STRiP	
Training	Coverage	Chr 11	Chr 20	Chr 11	Chr 20
Split	100%	95.03	94.39	92.15	90.03
	75%	94.57	93.43	91.76	89.90
	50%	94.01	91.97	90.81	88.18
	25%	86.86	84.39	84.62	80.20
Chrom	100%	93.17	92.51	92.15	90.03
	75%	92.78	91.49	91.76	89.90
	50%	92.78	91.49	90.81	88.18
	25%	86.01	83.28	84.62	80.20
Simulation	100%	92.39	90.39	92.15	90.03
	75%	92.02	90.07	91.76	89.90
	50%	91.56	89.97	90.81	88.18
	25%	85.62	82.47	84.62	80.20

We run GINDEL using the following training strategies. Split: train with 75% genotypes and call the rest 25%. Chrom: train with data from the other chromosome. Simulation: train with simulated data. Coverage: percent of reads from the original data used in genotype calling. Genotype accuracy is reported as the percentage of genotypes called correctly.

#### Genotypes for shorter deletions with low coverage real data

The results from [18] are mostly for longer deletions. And we did not find any real population sequence reads with benchmarked genotypes on short deletions. So in order to evaluate the performance of genotype calling on deletions of different lengths, we use an indirect way. We use the real sequence reads in the 1000 Genomes Project from the same 33 sequenced individuals in the high resolution CNV benchmark data from chromosome 11. For deletions, we use all the 737 reported deletions on the chromosome 11 that are released by the 1000 Genomes Project pilot phase (released at June 2010). Note that the 1000 Genomes Project did not release the genotypes for these deletions. In order to evaluate the genotype accuracy, we use the following strategy. We use SVseq2 [7] and Genome STRiP (only the Discovery part) to call deletions on reads from each of the 33 individuals. Note that we treat each individual at a step (i.e. not pooling reads from multiple individuals). SVseq2 and Genome STRiP call 1,106 and 510 deletions respectively. We then use the deletions called by both SVseq2 and Genome STRiP, which are also included in the released pilot data release from the 1000 Genomes Project. There are 267 such deletions. For each deletion, the genotype is marked as “no deletion” or “with deletion” for each individual. That is, we do not distinguish homozygous and heterozygous deletions. In particular, we mark a genotype of an individual to be “with deletion” if this deletion is called out by both SVseq2 and Genome STRiP for this individual. Similarly, if a deletion is neither called out by SVseq2 nor by Genome STRiP, it is marked as “no deletion”. To get more accurate genotype marking, deletions for cases where there are conflicts between SVseq2 and Genome STRiP are not used in comparison. We also test how read coverage affects the results by using a fraction of reads.

**Figure 3:**
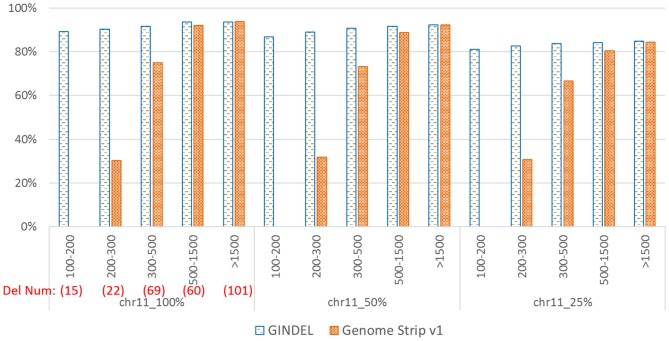
Comparison of GINDEL and Genome STRiP for calling deletion genotypes on real reads from the 1000 Genomes data on deletions of various sizes. Horizontal axis: range of deletion length (in bp). The number of deletions within a length range is given in the left part (below the length). Vertical axis: percentage of genotypes that agree with the genotype marking. We also give the number of deletions within each length range (in the left part, below the deletion length. For example, there are 15 deletions within range of 100 to 200 bp. Genome STRiP v1 does not call genotypes for deletions shorter than 200 bp, and so no result is given.

Figure 3 shows the results of GINDEL and Genome STRiP with different fraction of data used (100%, 50% and 25%). GINDEL is trained using simulated data. Note that the reported accuracy here is the percentage of the called genotypes matching the marked genotypes (i.e. if the called genotype is homozygous wild-type and the marking is “no deletion” or the called genotype is not homozygous wild-type and the marking is “with deletion”). One can see that GINDEL outperforms Genome STRiP in most cases, especially for the shorter deletions (400 bp or shorter). Note that there are many such shorter deletions: there are 101 such deletions (about 37.8% of all deletions). This is consistent with the results on simulated data in the “Results for different deletion sizes and coverages” Section. We also note that GINDEL performs well when different fraction of data is used.

#### Genotypes for deletions with high coverage trio data

To further evaluate the performance of GINDEL on real data and compare with Pindel and Clever-sv, we perform experiments on a group of high coverage trio data released by Illumina (Platinum Genomes, http://www.ebi.ac.uk/ena/data/view/ERP001960). Three samples NA12877 (father), NA12878 (mother), and NA12882 (son) are used, and the average coverage of each sample is about 50×. First, we run SVseq2, Pindel, and Mate-Clever to call deletions for these three samples on chromosome 11. The numbers of deletions called by SVseq2, Mate-Clever and Pindel are 94, 2,504 and 38,008 respectively. Second, we collect the 40 deletions that are called out by all three tools (using a slack value 5 bp on both end of a deletion when comparing the called deletions). Then, we call genotypes for these 40 deletions using GINDEL, Clever-sv and Pindel. For GINDEL, we use simulated data to train the models. Two datasets (6 samples for each) of coverages 50× and 10× are simulated, and we use the same 221 deletions used in the simulation section. Detailed information on training is provided in the Material S1. To evaluate the performance of tools on data with different coverages, we perform downsampling for the original data. To perform downsampling, samtools with option “view –s” is used, and two datasets are generated with average coverage 12.5× and 7.5× respectively. Note that Pindel cannot genotype deletions with given positions. That is, Pindel needs to start with deletion finding in order to call genotypes. So when coverage decreases, deletions called out may change to another genotype or sometimes not be called by a tool. In our experiments, for coverage 12.5× and 7.5×, 36 and 31 out of the 40 deletions are called out respectively by Pindel.

Three metrics are used to evaluate the performance of the tools.

1. The number of deletions violating Mendelian inheritance. Genotypes of trio can be checked for Mendelian inheritance. For example, if both parents have genotypes 2 at a deletion, then their child will almost always have genotype 2 at the same deletion. Eight discordant cases for the trio genotypes are used: (0,0,1),(0,0,2), (0,1,2), (0,2,0), (0,2,2), (1,2,0), (2,2,0), and (2,2,1), where the first two represent the genotype of the parents, and the third one is the genotype of the child. A discordant case is an indication that some called genotypes in the trio may be wrong.
2. The number of deletions with all-0 genotypes for the trio. Because the coverage is quite high, and the deletions are called out by all three tools, it is highly likely that the deletion allele appears in at least one family member of the trio for each of the 40 deletions. So the all-0 genotypes of the family at a deletion is an indication of genotype error in some of family members at the deletion.
3. The number of changed genotypes when coverage decreases. We assume that all methods perform better at higher coverages (e.g. 50×) than at lower coverages (e.g. 7.5×). Then when we perform downsampling, some genotypes called at lower coverage may be different (either a different genotype or not called) from those called at high coverage. These genotypes are likely to be wrong due to insufficient data for the particular method.

Table 6 shows the results of GINDEL, Pindel, and Clever-sv on the high coverage trio data. The results shows that, when coverage is at 50×, the results for GINDEL, Pindel, and Clever-sv are simliar. There are only one or two deletions that do not agree with Mendelian inheritance or have all-0 genotypes. This supports our assumption that all three tools perform well at high coverage. However, as the coverage decreases, Pindel performs less well. For example, at 12.5× coverage, 10 out of 36 deletions violate the Mendelian inheritance, and 5 out of 36 deletions with all-0 genotyps. Clever-sv appears to perform better than Pindel, especially at higher coverage. However, at lower coverage (say 12.5×), at least one genotype cannot be called out for half of the 40 deletions. In contrast, we notice that GINDEL performs relatively well as coverage reduces. For the worst case, only three deletions violate Mendelian inheritance. Moreover, GINDEL produces more consistent called genotypes when coverage reduces from 50× to 7.5×: the percentage of changed genotypes is 17.50% for GINDEL, 72.05% and 36.67% for Pindel and Clever-sv respectively.

**Table 6:** Comparison of GINDEL, Pindel and Clever-sv on high coverage trio data using downsampling.

Coverage	Metrics	GINDEL	Pindel	Clever-sv
100%(50×)	# discordant deletions	1(40)	1(40)	0(40)
	# deletions with all-0 genotypes	2(40)	1(40)	2(40)
25%(12.5×)	# discordant deletions	0(40)	10(36)	0(20)
	*#* deletions with all-0 genotypes	1(40)	5(36)	2(20)
	*#* changed genotypes in downsampling	11(120)	35(108)	30(120)
15%(7.5×)	# discordant deletions	3(40)	5(31)	0(14)
	*#* deletions with all-0 genotypes	1(40)	21(31)	1(14)
	*#* changed genotypes in downsampling	21(120)	67(93)	44(120)

The number of possibly wrongly called deletions (outside the parenthesis) and the total number of deletions (inside the parenthesis) are reported based on three creteria. # discordant deletions: number of deletions violating the Mendelian law; # deletions with all-0 genotypes: number of deletions with genotype 0 for both parents and the child in the trio (which contradicts the calling of the deletion from the same trio data); # changed genotypes in downsampling: the number of the genotypes that are called differently for the down-sampled data when compared with the full data results.

For coverage 50×, we also compare the concordance between GINDEL, Pindel and Clever-sv. The concordance ratio is 87.50% for GINDEL and Clever-sv, 86.67% for GINDEL and Pindel, and 84.17% for Pindel and Clever-sv.

### Insertion genotype calling with simulated data

GINDEL can also call genotypes of insertions. We test our method on both simulated and real data. For simulated data, 25 individuals based on human chromosome 11 of NCBI 37 are simulated. There are 126 insertions of chromosome 11, with the minimum size 33 bp, the maximum size 6,017 bp and the average size 282 bp, as released by the 1000 Genomes Project pilot phase. We randomly create the genotype for each insertion site with 10% homozygous insertions, 70% homozygous wild-type, and 20% heterozygous insertions. For heterozygous insertions, we arbitrarily put the insertions on one of the two copies. Reads are simulated in the same way as in the deletion case (explained as above). Here is the accuracy of called insertion genotypes by GINDEL. The percentages of accurately called genotypes are 94.38%, 92.10%, and 91.30% for coverage at 6.4×, 4.2× and 3.2× respectively.

### Insertion genotype calling with real data

To further evaluate the performance of GINDEL on insertion genotype calling on real data, we test on a group of high coverage trio data released by the 1000 Genomes Project at pilot 2 stage. Three samples NA12891 (father), NA12892 (mother), and NA12878 (child) are used with average coverage of about 40×. The positions and lengths of insertions are also released by the 1000 Genomes Project on June 2010 (contained in file CEU.trio.2010
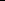
06. MobileElementInsertion.sites.vcf). We collect all the insertions larger than 50 bp called on chromosome 11, which include 66 insertions with minimum, median, and maximum length being 50 bp, 292 bp, and 6,062 bp respectively. We perform downsampling to evaluate the performance on data with different coverages. The “view –s” option of samtools is used to get 50% and 25% of the original data. We train the models on simulated data. Two datasets (6 samples for each) with coverage 40× and 15× are simulated based on chromosome 11. We use the simulated data whose coverage is 40× to train a model and then test on the real data with coverage 40×, and use simulated data with coverage 15× to train a model and then test on the other two datasets of real data. More detailed information on simulation and training is given in the Material S1.

Two out of the three metrics used in the deletion genotype calling for high coverage trio data are used here to evaluate the performance: the number of discordant insertions that violate the Mendelian law and the number of changed genotypes when coverage decreases. We do not use the other metric (the number of insertions with all genotypes are 0) because the 66 insertions are called out from 6 samples (two group of trio data), thus many deletions are only exist in the other trio data but not exist in the trio data used here, so it is normal to have a insertion that all the genotypes of the three samples are 0. Table 7 shows the detailed results of insertion genotype calling on different coverage data. The results show that GINDEL works well on both high and low coverage data.

**Table 7:** Performance of insertion genotype calling of GINDEL on high coverage trio data using downsampling.

Coverage	# of discordant insertions	# of changed genotypes
100%(40×)	3(66)	-
50%(20×)	3(66)	8(198)
25%(10×)	6(66)	21(198)

The number of possibly wrongly called insertions (outside the parenthesis) and the total number of insertions (inside the parenthesis) are reported based on two creteria. # discordant insertions: number of insertions violating the Mendelian law; # changed genotypes in downsampling: the number of the genotypes that are called differently for the down-sampled data when compared with the full data results.

### Running time and memory usage

GINDEL runs reasonably efficiently with less memory consuming on the data we test. As one example, we run GINDEL, Genome STRiP, Pindel and Clever-sv on simulated data from chromosome 15, where there are 6 individuals and 221 deletions with the average coverage 10×, and the server configuration is: eight core CPU(Intel(R) Xeon(R) ×5482 @ 3.20 GHz) with 32 G memory. The time for GINDEL is 105 minutes and 8 seconds (including training and testing), and the maximum memory usage is 1.5%. For comparison, Genome STRiP takes 201 minutes and 18 seconds for preprocessing and genotyping with maximum memory usage 7.2%, Pindel takes 3220 minutes and 37 seconds to call and genotype deletions with maximum memory usage 19.8%, and Clever-sv takes 1203 minutes and 39 seconds to do alignment and genotyping with maximum memory usage 37.8%. Recall that Pindel cannot call genotypes only at specified positions, and needs to call out the deletions first. And to get a good results, Clever-sv needs to re-align the raw reads to reference using a tool called laser which is time consuming.

## Discussion

**Figure 4:**
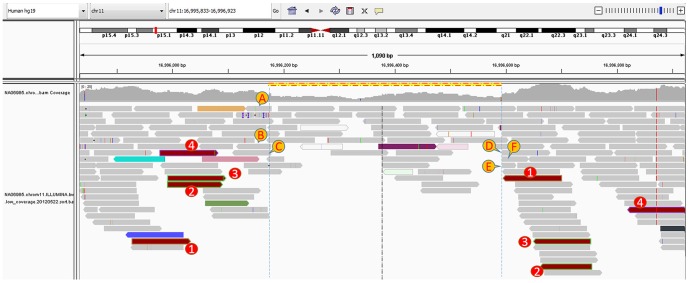
Sequence reads near a 425 bp deletion (marked as yellow dashed line) on chromosome 11. GINDEL calls the genotype 1 (which is more likely to be correct) while Genome STRiP calls 0. A,B,C,D,E,F: the mapped parts of six split-reads. 1, 2, 3, 4: four discordant read pairs on the left and right sides.

Overall, we show in this paper that GINDEL is more accurate and applicable than Genome STRiP, Pindel and Clever-sv for deletion genotype calling in the range of data we tested. We note that our simulation by no means covers all possible cases. GINDEL achieves higher genotype accuracy due to two improvements. First, GINDEL uses more features extracted from sequence reads. For example, GINDEL uses spanning reads that are not used by Genome STRiP, uses read depth that is not used by Clever-sv, and uses both discordant and concordant paired-end reads that are not used by Pindel. For insertion, GINDEL uses reads that are partially mapped. Since sequence data is usually noisy, using more information from the reads is important for accurate genotype calling. Second, GINDEL uses a machine learning approach in combining different features. This is different from other three tools. For example, Genome STRiP uses a Bayesian likelihood approach. Note that features (e.g. discordant encompassing pairs and split-reads) can vary greatly in their nature and determining how to weigh different features when calling genotypes may not be straightforward. GINDEL gets around this problem using a machine learning approach. For illustration, we show an example from the real low coverage 1000 Genomes Project data analyzed in the Results Section. The deletion is located on chromosome 11 from 16,996,169 to 16,996,593 (shown as yellow dashed lines). Both SVSeq2 and Genome STRiP find this deletion (also in the 1000 Genomes Project release) in the individual NA06985. Thus, this individual likely contains at least one deletion allele. Figure 4 shows the IGV [20] view of the reads near the deletion for individual. Reads marked as A, B, C, D, E, and F are the split-reads (only the mapped segments are shown). Read pairs marked as 1, 2, 3 and 4 are the discordant encompassing pairs. We can also see there are fully-mapped spanning reads, and the read depth is smaller than regions without indels but is still higher than 0. By combining all these features, GINDEL calls the genotype 1, which is likely to be correct based on the collected features. In contrast, Genome STRiP calls genotype 0 for this deletion (even when it finds this deletion in the discovery phase for this individual). This is because the likelihood computed by Genome STRiP for genotype 0 is slightly higher than that of genotype 1.

One issue of using GINDEL is that GINDEL needs training data (i.e. sequence reads with known genotypes). We expect that more validated benchmark data for indel genotypes may become available in the near future. In the case that no suitable training data is available, our results show that simulated data can also be used for training, and then the trained model can be used to call genotypes of real biological data. One should note that this approach is slightly less accurate than using real data in training. Thus, training with real data is recommended when such data is available. One advantage of separating training from genotype calling is that GINDEL can call single individual's genotypes using a trained model, while Genome STRiP does not work for single individual but requires multiple individuals' sequence reads even when only one individual's genotypes are to be called.

The approach used by GINDEL can in principle be extended to call genotypes of other types of structural variations. For example, with modification, GINDEL may be able to call genotypes of indels that are shorter than 50 bp. We may still be able to rely on multiple features extracted from sequence reads. For shorter indels, different features may be needed. In particular, encompassing discordant pairs may be less effective for shorter indels.

## Supporting Information

Material S1 **These files provide supplementary materials.**
(PDF)
