# Supplementary material for: GINDEL: Accurate Genotype Calling of Insertions and Deletions from Low Coverage Population Sequence Reads
Source: PLoS One. 2014 Nov 25;9(11):e113324. doi: 10.1371/journal.pone.0113324 (PMC4244156; doi:10.1371/journal.pone.0113324)
Supplement: Material S1 — These files provide supplementary materials. (PDF) [file pone.0113324.s001.pdf]

# GINDEL: accurate genotype calling of insertions and deletions from low coverage population sequence reads

Chong Chu [1], Jin Zhang [2] and Yufeng Wu [1*]
[1] Department of Computer Science and Engineering, University of Connecticut, Storrs, CT 06269, U.S.A.
[2] The Genome Institute, Washington University School of Medicine, St. Louis, MO, 63110, USA.

## Model training and genotype calling

GINDEL uses SVM to call genotypes of indels. So we need to train a model first, and then use the trained model to call genotypes. To train a model, users need to run GINDEL first to collect features, and then label each vector with the known (either validated or simulated) genotypes. Then to call genotypes, users also first need to run GINDEL to collect all the features, then using LibSVM to predict with the trained model.

### 1. Prepare training data

It is recommended to use validated real data to train a model, which will help to get better results. However, sometimes if there is no validated data available, we can train the model using simulated data.

1.1 Using validated data as training data

GINDEL provides a “-g” option to help to convert the output into the format that can be accepted by LibSVM. Users should provide the genotype information in vcf format.

1.2 Using simulated data as training data

If there is no validated data available, users can use simulated data to train a model. A simulator is released in our program (http://sourceforge.net/projects/gindel/). Go to the specification for more detailed information on how we generate haplotypes, sequence reads, and do alignment. To get better results, users are recommended to simulate data with the similar coverage, read length, mean insert size, standard deviation and error rates as the real data.

### 2. Train a model

To train a model, we use the command: “python easy.py data”, where “data” is the data for training. If the data is in the right format, then a model “data.model” will be generated.

### 3. Genotype calling with a trained model

To call genotype using the trained model, we use the command: “svm-predict predictData.scale Result.model predictResult”, where “Result.model” is the trained model, predictData.scale is the scaled data to be predicted, and “predictResult” is the finally called genotypes. To scale the data, users can use the “svm-scale” tool provided in LibSVM package.

## Command used to run the tools

We release all the scripts we use to run Genome Strip, Clever-sv, Pindel and Gindel on our program website (http://sourceforge.net/projects/gindel/).

## Results

### 1. Results of deletions

#### 1.1 Detailed results of simulation data.

The simulated data includes 45 individuals simulated from chromosome 15 of NCBI36. We introduce 221 deletions from the CEU population reported by Mills et el., 2011 to the simulated datasets. The deletion file is union.2010 06.deletions.genotypes.vcf.gz in the 1000 Genomes Project data release. For these deletions, the smallest deletion size is 51 bp, the largest deletion size is 160,798 bp, and the average size is 339 bp. Because the haplotypes are unknown, for heterogeneous deletions we arbitrarily put one copy of the deletion on one of the haplotypes of an individual. We simulate paired-end reads with the reads simulator wgsim, with 2% error rate, read length of 100, and insert size of 500. Three datasets with coverage 4.2x, 6.4x, and 10.0x are simulated. BWA is used with default parameters to map the simulated reads to NCBI36. The benchmark of simulated data is shown Table S1.

##### 1.1.1 Results of GINDEL of different deletion sizes and coverages

The results are shown in Table 1, Table S2, Table S3.

##### 1.1.2 Results of GINDEL of different deletion frequencies

The results are shown in Table 7 and Table S4.

##### 1.1.3 Results of GINDEL on different combined features

The results are shown in Table 3 and Table S5.

##### 1.1.4 Results of GINDEL of training and testing on different chromosomes

We train a model by using data simulated from chromosome 15, and test on data simulated from chromosome 20. The results are shown in Table S6.

##### 1.1.5 Results of Genome STRiP on different deletion sizes and coverages

The results are shown in Table 1 and Table S7.

##### 1.1.6 Results of Clever-sv on different deletion sizes and coverages

The results are shown in Table 1 and Table S8.

### 1.1.7 Results of Pindel on different deletion sizes and coverages

The results are shown in Table 1 and Table S9.

## 1.2 Detailed results of real data.

### 1.2.1 Results of High resolution CNV benchmark data

We first use the low-coverage population sequence Phase two data released by the1000 Genomes Project. This data consists of alignment files of 66 individuals on chromosomes 11 and 20 (evenly distributed for each chromosome). For the 33 individuals of each chromosome, 13 of the individuals are from CEU population and the others are from the YRI population. These alignment datasets are mapped using BWA with soft-clips on NCBI37. There are 122 deletions in these two chromosomes, where genotypes are called in Conrad et al., 2010. These CNVs are validated through array-CGH using a set of NimbleGen, and thus we use these genotypes as our benchmark. These deletions are usually long: only 16 are within the range of 450 bp to 960 bp, and the rest others are longer than 1 kbp. The minimum deletion size is 450bp. The maximum deletion size 88,384 bp, and average size 2,461 bp. To evaluate GINDEL and Genome STRiP for data with different coverages, we manipulate the reads by randomly keeping a fraction of the reads in the original data. The fractions are chosen from 100% (i.e. using all the reads), 75%, 50% and 25%. The total number of genotypes are shown in Table S10.

For GINDEL, because we can train a model in different ways, thus several groups of comparison are done:

**1) Training and testing using data from same chromosome.**

Results are shown in Table 4, Table S11, and Table S12.

**2) Train with given genotypes on one chromosome, and use the trained model to call genotypes on the other chromosome.**

Results are shown in Table 4 and Table S13.

**3) Train with simulated data, and test on real sequence reads**

Results are shown in Table 4 and Table S14.

For Genome STRiP, the detailed results are shown in Table S15.

### 1.2.2 Genotypes for shorter deletions with real data

Detailed results are shown in Table S16.

## 1.3 Results of high coverage trio data

Detailed results of GINDEL, Clever-sv, and Pindel are shown in Table 5.

## 2. Results of insertions

### 2.1 Results of simulated data

Detailed results are shown in Table S17.

### 2.2 Results of high-coverage trio data

Detailed results are shown in Table 6.
